# Artificial Creativity: from predictive AI to Generative System 3

**DOI:** 10.3389/frai.2025.1654716

**Published:** 2025-10-15

**Authors:** Juan Carlos Chávez-Autor

**Affiliations:** ^1^College of Psychology, Keiser University, Fort Lauderdale, FL, United States; ^2^Facultad de Comunicación, Universidad Panamericana, Mexico City, Mexico; ^3^School of Business, Economics and Law, University of Gothenburg, Gothenburg, Sweden; ^4^Bio-Intelligence and Creativity Institute, Playa del Carmen, Mexico

**Keywords:** Artificial Creativity, Generative System 3 (GS-3), large language models (LLMs), adaptive gain control, computational creativity, System 3 (metacognitive control), exploration–exploitation trade-off, tri-process cognition

## Abstract

Large language models generate fluent text yet often fail to sustain novelty, task relevance, and diversity across extended contexts. We argue this shortfall persists because current systems implement only fragments of a tri-process loop that supports human creativity: spontaneous ideation in the default-mode network (DMN; broadly *System 1*–like), goal-directed evaluation in the central-executive network (CEN; broadly *System 2*–like), and a metacognitive integrator—*System 3*—that, via neuromodulatory gain control, shifts between exploration and focused control. We introduce *Generative System 3* (GS-3), an architecture-agnostic design pattern with three roles: a high-entropy *generator*, a learned *critic*, and an adaptive *gain controller*. Beyond “pure prediction” and simple “reflective prompting,” GS-3 identifies the missing pieces for *Artificial Creativity*: an internal evaluator, endogenous control over sampling entropy, and adaptive priors maintained across extended contexts. This conceptual analysis (i) formalizes *novelty*, *usefulness*, and *diversity* with operational definitions; (ii) develops multiple gain-update policies (exponential, linear, logistic) with stability constraints and sensitivity expectations; (iii) derives falsifiable behavioral indices—*associative-distance density*, *analytic-verification ratio*, and *convergence latency*—with pass–fail criteria; and (iv) provides a proof-of-concept blueprint and evaluation protocol (tasks, metrics, ablations, reproducibility kit). We position GS-3 relative to computational-creativity and co-creative frameworks, and delineate where brain–model analogies are functional rather than literal. Ethical guidance addresses bias, cultural homogenization, and reward gaming of proxy objectives (often termed “dopamine hacking”) through plural critics, transparent logging, and outcome-tied entropy caps. The result is a testable roadmap for transitioning from regulated prediction to genuinely creative generative systems.

## 1 Introduction—from fluent prediction to creative control

Large language models (LLMs) now produce remarkably fluent text, yet they often struggle to sustain novelty, task relevance, and diversity across extended contexts. We argue this shortfall persists because current systems implement only fragments of a tri-process loop that supports human creativity: spontaneous ideation in the default-mode network (DMN; broadly aligned with *System 1*), goal-directed evaluation in the central-executive network (CEN; broadly aligned with *System 2*), and a metacognitive integrator—*System 3*—that, supported by neuromodulatory gain, shifts the mind between exploration and focused control to achieve integrative creative outcomes. In this view, dual-process accounts are necessary but not sufficient; System 3 coordinates and regulates how ideas are generated and pruned, turning “thoughts of thoughts” into adaptive action. The convergence of these mechanisms in machine systems is what we call *Artificial Creativity*.

### 1.1 The missing link between predictive AI and creative cognition

From a neuroscience vantage, creative performance depends on flexible DMN–CEN interaction, with dopaminergic signals modulating the exploration–exploitation balance (Chen et al., 2025; Shine, 2019; Westbrook et al., 2021). By contrast, most LLMs behave like DMN-only decoders: excellent at sequence extension, but lacking an internal evaluator and endogenous gain control to decide when to broaden or narrow the search. Bridging this gap requires importing System 3 principles into model design.

### 1.2 Why a conceptual analysis now?

Evidence on both sides is converging. LLMs can match or exceed median human fluency on some divergent-thinking tasks, yet at scale, their outputs tend to homogenize, reducing collective diversity (Doshi and Hauser, 2024). Human–AI co-creation increases speed and fluency but, without structure, can dampen variety or drift from task goals (Chen and Chan, 2024; Chakrabarty et al., 2024). Meanwhile, covert neurofeedback that strengthens DMN–CEN coupling elevates originality in human participants (Luchini et al., 2025). Together, these findings motivate a synthesis that links cognitive theory, neural evidence, and generative-model engineering—and states testable criteria for when an artificial system merits the label creative. Throughout, any brain–model correspondences are treated as functional analogies, not biological isomorphisms.

### 1.3 Contribution and scope

This conceptual analysis does not report new empirical data. Instead, it advances a falsifiable framework—*Generative System 3* (GS-3)—and a concrete evaluation program. GS-3 is an architecture-agnostic design pattern with three roles: a high-entropy *generator* (idea expansion), a learned *critic* (context-sensitive appraisal), and an adaptive *gain controller* (endogenous regulation of sampling entropy). We contribute four elements: (i) operational definitions of *novelty* (distributional distance to a baseline), *usefulness* (task-conditioned utility), and *diversity* (across-run dispersion); (ii) behavioral indices with pass–fail criteria—*associative-distance density*, *analytic-verification ratio*, and *convergence latency*—so the theory can be falsified in practice; (iii) a mathematical treatment of gain policies (exponential, linear, logistic) with stability constraints and sensitivity expectations; and (iv) a proof-of-concept blueprint and evaluation protocol (tasks, metrics, ablations, reproducibility kit) that research groups can implement.

We situate these contributions along a predictive-to-generative continuum. *Pure prediction* extends sequences without internal evaluation. *Regulated generation* introduces external controls (e.g., temperature, top-*k*) but still lacks an inner judge. *Reflective generation* uses self-prompted critique yet remains scaffold-dependent. *GS-3–level creativity* emerges only when a system (a) cycles autonomously between idea expansion and evaluative pruning, (b) adjusts its own sampling entropy in response to real-time reward-prediction error, and (c) maintains adaptive priors over long contexts.

### 1.4 Roadmap

Section 2 positions GS-3 within computational-creativity traditions, LLM-based co-creation, and alternative theories. Section 3 summarizes the DMN/CEN/dopamine template and its limits (functional analogies, not isomorphisms). Section 4 presents the GS-3 architecture with formal definitions, falsification tests, and gain-policy mathematics. Section 5 offers a proof-of-concept blueprint and evaluation protocol (hypotheses, metrics, ablations). Section 6 locates today’s systems on the continuum and identifies gaps GS-3 fills. Section 7 expands ethics and governance with concrete mitigation steps. Section 8 concludes with a Discussion that synthesizes contributions, states boundary conditions, and outlines future work. By unifying cognitive theory, network neuroscience, and AI engineering, we aim to establish Artificial Creativity as a testable construct and to provide a practical roadmap for building and auditing genuinely creative generative systems.

## 2 Computational creativity traditions

Early taxonomies emphasize what counts as creative behavior and how to evaluate it. Boden’s distinctions between psychological and historical creativity (P- vs. H-creativity) foreground the mechanisms of combinational, exploratory, and transformational search (Boden, 2004). Formal accounts characterize creative systems by their generative space and constraints (Wiggins, 2006), while evaluation frameworks propose measurable criteria for attributing creativity to artifacts or systems (Ritchie, 2007; Jordanous, 2012). System exemplars, such as The Painting Fool, demonstrate end-to-end pipelines that produce artifacts and internal justifications (Colton, 2012).

These traditions supply two pillars we retain: (a) creativity requires both a generator of candidates and a process that evaluates them in context, and (b) claims should be tied to operational criteria. GS-3 extends this foundation by adding an explicit, adaptive gain mechanism that regulates the breadth/depth of search online and by specifying falsifiable behavioral indices (novelty, usefulness, diversity) together with pass/fail thresholds. In short, GS-3 preserves the generator–evaluator logic but makes the regulator a first-class component with its own dynamics.

### 2.1 LLM creativity and co-creation

Modern LLM pipelines span a pragmatic continuum. *Pure prediction* extends sequences using maximum-likelihood training (Sutskever et al., 2014). *Regulated generation* introduces external controls—temperature and top-*k*/top-*p* settings shape randomness, and beam search maintains several high-probability continuations—which can prevent degeneracy but do not install an inner judge (Holtzman et al., 2020). Steering methods alter token probabilities directly (e.g., plug-and-play controls for attributes without backbone retraining) (Pascual et al., 2021). *Reflective prompting* scaffolds brief internal critique (e.g., chain-of-thought) and retrieval-augmented generation injects external knowledge to improve coherence and factuality (Chu et al., 2024; Izacard and Grave, 2021). *Self-improvement/self-correction loops* iteratively revise drafts with model feedback (Kamoi et al., 2024; Ding et al., 2024). *Multi-agent set-ups* coordinate multiple LLMs to critique and debate (e.g., generative agents) (Park et al., 2023).

Empirically, co-creation studies show that LLM support often increases fluency and speed but can reduce variety without structured collaboration protocols (Chen and Chan, 2024; Chakrabarty et al., 2024). At the population scale, assistance can increase individual originality while decreasing collective diversity, consistent with homogenization risks (Doshi and Hauser, 2024). Conceptually oriented analyses debate whether current LLMs meet creativity criteria and where limits remain (Franceschelli and Musolesi, 2024; Floridi and Chiriatti, 2020).

GS-3 aligns with these trajectories yet differs on one decisive point: the evaluator and the regulator are internal, learned, and adaptive. Rather than relying on hand-tuned temperatures, prompt engineering, or fixed debate scripts, GS-3 requires a critic that computes task-conditioned utility and a gain controller that adjusts sampling entropy based on reward-prediction error. This makes the exploration–exploitation balance endogenous to the system and testable via ablations (remove critic or gain; swap update rules; vary the learning rate).

### 2.2 Complementary theories and boundary conditions

Information-theoretic and intrinsic-motivation accounts explain why systems seek novelty or compressive structure (Schmidhuber, 2010). Evolutionary approaches operationalize open-ended novelty (Lehman and Stanley, 2011). Predictive-processing and free-energy views model perception and action as minimizing prediction error or free energy under learned priors (Clark, 2013; Friston, 2010). These perspectives illuminate why creative systems might alternate between broad exploration and tight verification.

GS-3 is compatible with these theories but adds a concrete control story: a generator produces candidates; a critic scores them relative to task and context; and a gain controller adjusts entropy and effort in real time, producing measurable signatures (e.g., shifts in associative-distance density and verification ratios). Where embodied and enactive views stress sensorimotor grounding, GS-3 can be seen as the cognitive-control core that any grounded agent still requires to manage the breadth and depth of search. Where information-theoretic approaches prize compression or novelty alone, GS-3 foregrounds usefulness by design through the critic’s utility function. Finally, where predictive-processing emphasizes error minimization, GS-3 specifies when and how the system should temporarily widen its hypothesis space before re-engaging verification.

Together, these comparisons place GS-3 as a synthesis that retains the generator–evaluator insight from computational creativity, adopts practical controls from contemporary LLM pipelines, and formalizes the missing adaptive regulator. Subsequent sections develop the architecture (Section 4), metrics, and gain policies with falsification tests (Section 4), and outline a proof-of-concept and evaluation protocol suitable for empirical validation (Section 5).

## 3 Neurobiological template

Creativity does not reside in a single cortical locus; it emerges from interactions among large-scale networks modulated by neuromodulatory systems. In broad terms, associative expansion is linked to the default-mode network (DMN), and evaluative control is associated with the central-executive network (CEN), while neuromodulators, such as dopamine, bias the system toward exploration or exploitation by altering integration and segregation dynamics. This section summarizes key findings and clarifies where brain–model analogies are functional (useful for design) rather than literal (biological identity).

### 3.1 Large-scale network architecture: DMN–CEN coupling

Resting-state and task-based studies converge on a picture in which creative performance is associated with flexible interaction between DMN hubs (e.g., medial prefrontal, posterior cingulate, temporoparietal regions) and CEN hubs (e.g., dorsolateral prefrontal, posterior parietal cortex). Using state-transition analyses of fMRI during divergent thinking, dynamic switching between DMN and executive-control states predicts higher originality and richer associative distance, consistent with the idea that creativity benefits from alternating expansion and evaluation rather than the dominance of either mode alone (Chen et al., 2025). This dynamic view situates creativity as a property of network coupling over time, not a static activation pattern.

### 3.2 Neuromodulatory gain and the exploration–exploitation balance

Neuromodulatory accounts propose that large-scale brain dynamics shift between more integrated and more segregated network configurations as a function of arousal-linked chemical signals. Reviews of integration–segregation emphasize that noradrenergic projections from locus coeruleus and cholinergic projections from basal forebrain are prominent levers for these state transitions: modest changes in their tone can reconfigure connectivity, biasing cognition toward either broad, globally integrated processing or more locally segregated, task-focused processing (Shine, 2019). In parallel, work on dopamine and cognitive control links striatal D2 receptor availability to the subjective cost of exerting control and to cost–benefit decisions about engaging effortful processing, consistent with a role for dopamine in setting how deeply and persistently goal-directed search is pursued (Westbrook et al., 2021).

Together, these strands motivate an operational notion of gain: a control signal that widens or narrows the currently active hypothesis space. In integrated, high-gain states, the system samples more broadly (facilitating associative expansion); in segregated, low-gain states, it narrows and stabilizes processing (facilitating focused evaluation). We treat this as a functional template rather than a one-to-one biological mapping: multiple neuromodulators contribute to these shifts (Shine, 2019), and dopamine’s role is context dependent and tied to effort-related control policies (Westbrook et al., 2021). In the Generative System 3 framework, the abstract gain controller corresponds to an endogenous mechanism that adjusts sampling entropy online (e.g., via temperature), thereby implementing the exploration–exploitation trade-off that, in brains, is jointly shaped by neuromodulatory systems.

### 3.3 Multiscale evidence: genetics, oscillations, and causal perturbation

Evidence for individual differences in creative cognition appears across levels of analysis. At the macroscale, large-cohort multimodal work shows that a neural pattern predicting divergent-thinking performance carries positive weights in default-mode and frontoparietal control networks and is linked to dopamine-related neurotransmitters and genes influencing neurotransmitter release, indicating a biological substrate for variability in network dynamics relevant to creativity (Liu et al., 2024). At faster timescales, electrophysiological reviews report pre-solution modulations in low-frequency rhythms consistent with inwardly directed attention (alpha/theta changes) and brief gamma-band bursts localized to right anterior temporal cortex around the moment of insight—together aligning with a generate-then-verify sequence (Kounios and Beeman, 2014). Crucially, causal manipulations move beyond correlation: covert real-time fMRI neurofeedback that reinforces coactivation of default-mode and executive-control circuitry increases originality on divergent-thinking tasks relative to control conditions (Luchini et al., 2025). Collectively, these findings support a cycle in which associative expansion and focused appraisal are coordinated by state-dependent control signals, providing a biologically grounded template for the alternation mechanisms formalized in GS-3.

### 3.4 Translational lessons for artificial systems

Three design lessons follow for artificial systems seeking sustained novelty, usefulness, and diversity. First, at least two separable but re-entrant processing streams are required: a generator specialized for associative expansion and a critic specialized for task-conditioned evaluation. Second, a gain mechanism must adaptively regulate the breadth of search online; in practice, this means endogenously adjusting sampling entropy or effort as a function of a learned utility signal, rather than relying solely on fixed external controls. Third, the system should exhibit measurable signatures of alternation between expansion and verification over time. These lessons translate into testable predictions for Generative System 3: removing the critic should collapse usefulness at a fixed diversity level; removing the gain controller (freezing temperature) should eliminate alternation in associative-distance density and reduce across-run diversity, with within-run spread determined by the static decoding setting rather than by context; and reinforcing generator–critic coactivation (e.g., by rewarding alternation) should increase originality without sacrificing task relevance.

### 3.5 Boundary conditions and non-isomorphism

The DMN–CEN–dopamine template is a functional analogy, not a biological isomorphism. Biological networks operate with spiking dynamics, heterogeneous cell types, and complex neurochemical interactions; artificial networks are discrete symbol or vector processors trained under engineered objectives. Dopamine’s roles are multifaceted and context dependent, extending beyond a simple exploration knob; likewise, temperature in a language model is only one of several ways to regulate uncertainty. The analogy is therefore limited to architectural roles and control functions: generator versus evaluator interactions and an adaptive gain that shifts the exploration–exploitation balance. Our use of these mappings is pragmatic—intended to generate falsifiable design claims—rather than a claim of mechanistic identity.

## 4 GS-3 architecture: definitions, dynamics, and falsifiability

This section formalizes Generative System 3 (GS-3) while keeping implementation choices flexible. It specifies roles and interfaces, operational metrics, a bounded gain policy tied to a learning signal, behavioral indices with pass–fail criteria, and ablations. Full task lists, hyperparameters, and pseudocode remain in Supplementary Data Sheet 1.

### 4.1 Roles and interfaces

The architecture comprises three roles with explicit interfaces.

#### 4.1.1 Generator (G)

Proposes *k* candidates given a context, with a controllable sampling entropy (temperature *T*₍g₎). Output: a set of candidate continuations with scores from the base model (e.g., log probabilities).

#### 4.1.2 Critic (C)

Scores each candidate *x* with a task-conditioned utility *U*₍task₎ (*x*|task, context) ∈ [0, 1], returning a real-valued score for each candidate and the index of the winner. The critic may be a rubric-based classifier or a preference model trained from human feedback; in the latter case, its design, data provenance, and validation should follow published guidance on feedback-driven NLG (Fernandes et al., 2023; Casper et al., 2024).

#### 4.1.3 Gain controller (D)

Adjusts *T*₍g₎ online as a function of recent reward-prediction error, using a bounded policy (Section 4.3) and a smoothed baseline of expected utility to calibrate expectations. Output: the next-step temperature *T*₍g, new₎.

Minimal message flow per cycle is G → C → D → G, enabling alternating expansion and verification. This differs from externally tuned decoding (e.g., temperature sweeps or beam settings) and from pipelines that rely only on training-time preference alignment; here, usefulness is estimated by a learned critic active at inference time (Fernandes et al., 2023; Casper et al., 2024). Regulated decoding still matters—temperature/top-*p*/beam settings mitigate degeneracy (Holtzman et al., 2020)—but GS-3 requires an endogenous controller that adapts these levers during generation.

### 4.2 Operational definitions: novelty, usefulness, diversity

To permit falsification and fair comparison, we adopt simple, model-agnostic definitions.

#### 4.2.1 Novelty

Represent an artifact *x* (e.g., a paragraph) with an embedding *e*(*x*) from a fixed, publicly documented encoder. Relative to a preregistered baseline corpus, novelty increases as the nearest neighbor to *x* becomes more distant in cosine space (the exact nearest-neighbor formula appears in Supplementary Data Sheet 1).

#### 4.2.2 Usefulness

*U*₍task₎ (*x*) is a task-conditioned score in [0, 1], produced either by a rubric-based human panel or by a separately validated reward model trained on task-specific preferences (Fernandes et al., 2023; Casper et al., 2024). For experiments, preregister rubrics, rater training, and inter-rater reliability; when using reward models, report validation against held-out human judgments.

#### 4.2.3 Diversity

For a fixed prompt, report dispersion across independent runs (mean pairwise embedding distance). Exact formulas and encoder details are provided in Supplementary Data Sheet 1.

Together, novelty, usefulness, and diversity summarize originality, appropriateness, and dispersion and should be reported with confidence intervals.

### 4.3 Bounded gain policy and learning signal

Define the reward-prediction error as *δ*ₜ = *U*₍best, t₎ − *Ū*ₜ, where *U*₍best, t₎ is the critic’s top score at cycle *t* and *Ū*ₜ is an exponentially weighted moving average of recent best scores (full expression in Supplementary Data Sheet 1). The controller updates sampling temperature with a bounded logistic rule: *T*₍g₎(*t* + 1) = *T*₍min₎ + (*T*₍max₎ − *T*₍min₎)⋯*σ*(*α* + *η*·*δ*ₜ), where σ is the logistic function, η is a small learning-rate constant, and [*T*₍min₎, *T*₍max₎] are preregistered bounds. This yields smooth, monotone adjustments and prevents runaway entropy. Linear and exponential alternatives, stability notes, and sensitivity sweeps appear in Supplementary Data Sheet 1. The interpretation is consistent with accounts in which cost–benefit control policies regulate effort allocation, while remaining an engineering—not biological—control law (Westbrook et al., 2021).

Intuitively, each cycle compares the current best score to a smoothed baseline to obtain a “surprise” signal *δ*. If performance is better than expected (*δ* > 0), temperature increases smoothly; if worse (*δ* < 0), it decreases. Logistic squashing keeps *T*₍g₎ within preregistered bounds, making adjustments gradual and stable. The intercept α sets the default temperature when on trend, and the learning rate η controls how strongly surprises move it.

### 4.4 Behavioral indices and pass–fail criteria

#### 4.4.1 Associative-distance density (ADD)

Distribution of cosine distances between successive idea units within a run (e.g., sentences or design sketches). GS-3 prediction: alternating wide–narrow patterns reflecting expansion–verification cycling; regulated baselines: unimodal, temperature-dependent spread.

#### 4.4.2 Analytic-verification ratio (AVR)

Proportion of cycles in which C vetoes G’s top candidate and requests resampling at a lower *T*₍g₎. GS-3 prediction: AVR adapts to task difficulty; regulated baselines: AVR is fixed by external settings.

#### 4.4.3 Convergence latency (CL)

Cycles to meeting a preregistered success criterion (e.g., rubric score ≥ *τ*). GS-3 prediction: CL decreases within a session as *Ū* calibrates; reflective baselines show little within-session change.

#### 4.4.4 Pass–fail criteria

Preregister that a GS-3 system must (a) exceed a temperature-matched baseline on usefulness at equal novelty (dominance on the novelty–usefulness frontier), (b) achieve higher across-run diversity without external temperature sweeps, and (c) exhibit AVR and ADD signatures consistent with alternating exploration–verification (e.g., significant periodicity by spectral analysis). Computation details appear in Supplementary Data Sheet 1.

### 4.5 Formal hypotheses and ablation tests

H1 (critic necessity). Removing C (scores replaced by random or constant) reduces usefulness at matched novelty, collapsing the novelty–usefulness frontier.

H2 (gain necessity). Freezing *T*₍g₎ (no D) eliminates ADD alternation and reduces across-run diversity; usefulness becomes more sensitive to the initial temperature setting.

H3 (policy sensitivity). Logistic-, linear-, and exponential-gain policies occupy distinct regions of the novelty–usefulness–diversity space; logistic yields the best stability at comparable usefulness.

H4 (memory horizon). Increasing *β* (longer *Ū* memory) improves long-horizon coherence (e.g., cross-paragraph consistency) but slows adaptation after regime shifts.

Each hypothesis is falsifiable by implementing the corresponding ablation and reporting preregistered metrics with confidence intervals and effect sizes.

### 4.6 Design space and non-isomorphism

G and C need not be separate models; they may be two modes of a single backbone, two cooperating agents, or a backbone plus a lightweight preference head (as in instruction-following systems informed by human feedback; Fernandes et al., 2023; Casper et al., 2024). Likewise, D can be a small network conditioned on context features. Brain terms remain functional analogies: temperature is one of several levers (others include top-*k*/top-*p*, repetition penalties, and plug-and-play attribute controls) (Pascual et al., 2021). Retrieval can be added as an optional module to ground candidates; to avoid confounds, use the same retriever across GS-3 and RAG baselines (Izacard and Grave, 2021). The contribution here is to require that some endogenous gain exists, that it is coupled to a learned utility, and that its process-level signatures are measurable.

### 4.7 Implementation notes and comparators

For completeness and parity, report the decoding settings (temperature/top-*p*/beam) and sampling budgets for all conditions, including pure prediction (Sutskever et al., 2014) and regulated decoding baselines (Holtzman et al., 2020). When including reflective prompting as a comparator, preregister the exact scaffolds (e.g., chain-of-thought prompts) to ensure fair budgets and to acknowledge that such reflectivity remains externally scaffolded (Chu et al., 2024). If plug-and-play steering is used as a comparator, cite its peer-reviewed formulation and disclose active attribute controls (Pascual et al., 2021).

### 4.8 Interim summary

GS-3 embeds a learned critic and a bounded, adaptive gain policy into the generation loop, evaluated with preregistered novelty, usefulness, and diversity metrics plus cycling signatures. These commitments turn a functional analogy into a testable engineering target while remaining agnostic to backbone choice and compatible with standard comparators such as RAG, plug-and-play steering, and reflective prompting (Izacard and Grave, 2021; Pascual et al., 2021; Chu et al., 2024).

## 5 Proof-of-concept blueprint and evaluation protocol

This section describes how to implement and test a minimal instance of Generative System 3 (GS-3), specifying tasks, metrics, ablations, and analysis plans that allow other groups to falsify or support the framework.

### 5.1 Minimal implementation blueprint

#### 5.1.1 Architecture

Use a single transformer backbone with two heads: a generator head for next-token prediction and a critic head that outputs a task-conditioned utility score *U*(*x*|task, context). A lightweight controller maps recent reward-prediction error *δ* to an updated sampling temperature *T*₍g₎ for the next generation step (see Section 4 for definitions and bounds). This single-backbone design enables shared representations while keeping roles separable for ablations.

#### 5.1.2 Training the critic

Collect paired or graded preferences for task outputs using a rubric aligned with usefulness (e.g., goal fit, coherence, constraint satisfaction). Train the critic with supervised regression to predict human utility or with a preference model trained on pairwise comparisons, as in established human-feedback pipelines and surveys of feedback integration (Casper et al., 2024; Fernandes et al., 2023). Keep evaluation sets disjoint from critic training data.

#### 5.1.3 Controller policy

Implement the bounded logistic gain policy described in Section 4 as the default; include linear and exponential variants in preregistered sensitivity analyses with clipped *δ* and bounded *T*₍g₎. Preregister hyperparameter ranges and stopping rules.

#### 5.1.4 Baselines

Include three baselines: (a) pure prediction at multiple fixed temperatures; (b) regulated generation with beam search and temperature sweeps (Holtzman et al., 2020); and (c) reflective prompting (e.g., chain-of-thought) without an internal learned critic, using a recent survey as the canonical reference (Chu et al., 2024). Optional augmented baselines include retrieval-augmented generation using a published retrieval-and-generation pipeline (Izacard and Grave, 2021) and iterative self-refinement (Ding et al., 2024; see also the self-correction survey, Kamoi et al., 2024).

### 5.2 Tasks and datasets

#### 5.2.1 Divergent thinking

Adapt the Alternate Uses Test (AUT) to text prompts (e.g., “unusual uses for a paperclip”), as used in neuroimaging work on creative switching, to allow comparison with network findings (Chen et al., 2025). Score usefulness with a task rubric (plausibility under physical constraints), and compute novelty and diversity as in Section 4.

#### 5.2.2 Constrained creation

Short-form tasks, such as product names or headlines with explicit constraints (length, audience, and semantic cues), probe the critic’s ability to trade novelty for goal fit. Retrieval-augmented variants test whether GS-3 maintains benefits when external knowledge is available (Izacard and Grave, 2021).

#### 5.2.3 Long-horizon composition

Multi-paragraph story or concept-expansion tasks assess maintenance of adaptive priors and coherence over extended contexts. Include checkpoints for mid-course critique and revision.

#### 5.2.4 Human–AI co-creation

Writer-in-the-loop tasks mirror professional workflows and enable analysis of fluency–variety trade-offs (Chen and Chan, 2024; Chakrabarty et al., 2024).

#### 5.2.5 Population-scale dispersion

To test homogenization risk, elicit many outputs per prompt and quantify across-run diversity and mode collapse, following concerns documented at scale (Doshi and Hauser, 2024).

### 5.3 Metrics, reliability, and statistical analysis

#### 5.3.1 Primary metrics

Use the operational definitions from Section 4: novelty *N* (embedding distance from a baseline corpus), usefulness *U*₍task₎ (rubric or held-out reward model), and diversity *D* (mean pairwise distance across runs). Report within-run novelty and across-run diversity.

#### 5.3.2 Behavioral signatures

Compute associative-distance density (ADD) within runs, analytic-verification ratio (AVR; critic veto rate with resampling), and convergence latency (CL; cycles to reach a preregistered usefulness threshold). Assess periodicity in ADD to detect expansion–verification alternation.

#### 5.3.3 Reliability

For human scoring, report inter-rater reliability (e.g., Krippendorff’s alpha) and provide rater training materials. For model-based utility, validate the reward model against human judgments on a held-out set.

#### 5.3.4 Statistical plan

Preregister hypotheses, metrics, and analysis. Use hierarchical models or mixed-effects regressions to account for prompt and rater as random factors. Report effect sizes with confidence intervals and correct for multiple comparisons where applicable. Provide power analyses for planned contrasts (e.g., GS-3 vs. regulated baseline on usefulness at matched novelty).

### 5.4 Ablations and sensitivity

#### 5.4.1 Critic removal (H1)

Replace *U* with random or constant scores and re-run; predict collapse of usefulness at matched novelty.

#### 5.4.2 Controller freeze (H2)

Hold *T*₍g₎ constant; predict reduced across-run diversity and loss of ADD alternation.

#### 5.4.3 Policy comparison (H3)

Swap logistic (default), linear, and exponential policies while holding other components fixed; predict distinct novelty–usefulness–diversity trade-offs and fewer *T*₍g₎ saturations for logistic.

#### 5.4.4 Memory horizon (H4)

Vary *β* in the baseline *Ū*; predict improved long-horizon coherence at higher *β* but slower adaptation to shifts.

#### 5.4.5 Prompt perturbations

Vary prompt structure, length, and constraints to test robustness of gains. Include retrieval toggles to assess interaction with external knowledge (Izacard and Grave, 2021).

### 5.5 Reproducibility kit

Release code, model checkpoints (where licensing permits), exact prompts, rubrics, and analysis scripts. Fix random seeds; log *T*₍g₎, *δ*, *U*₍best₎, and *Ū* at each cycle for every run. Provide an audit sheet documenting compute budgets, training data used for the critic, and any human-in-the-loop procedures. For closed models, supply reproducible API settings and a synthetic variant using an open backbone.

### 5.6 Risk controls and fairness checks

#### 5.6.1 Homogenization audits

Track across-run diversity as a function of controller policy and dataset domain; include plural critics trained on diverse preference data to reduce mode collapse (Doshi and Hauser, 2024).

#### 5.6.2 Bias and equity

Stratify usefulness and novelty by dialect, register, or cultural domain. If disparities emerge, retrain or reweight critic data and re-test.

#### 5.6.3 Overfitting to graders

When using reward or preference models, separate training, validation, and evaluation distributions; periodically cross-check with human ratings to prevent exploitation of grader idiosyncrasies (Casper et al., 2024; Fernandes et al., 2023).

#### 5.6.4 Safety valves

Bound *T*₍g₎, clip *δ*, and cap cumulative entropy increases per session to prevent runaway exploration, consistent with concerns about degeneration under unbounded sampling (Holtzman et al., 2020).

### 5.7 Decision rule

Declare GS-3 support only if, on preregistered tasks, the system (a) dominates regulated and reflective baselines on usefulness at matched novelty, (b) achieves higher across-run diversity without external temperature sweeps, and (c) exhibits cycling signatures in ADD and AVR consistent with alternating expansion and verification. Otherwise, the framework is falsified for that task setting, and ablations should identify which component failed to contribute.

## 6 Positioning current systems on the predictive → generative continuum

This section locates prominent families of large language model (LLM) systems on a continuum from fluent prediction to partially reflective pipelines, and clarifies what each already achieves relative to Generative System 3 (GS-3). The emphasis is on the presence or absence of three ingredients that GS-3 treats as necessary for Artificial Creativity: (i) a generator capable of associative expansion, (ii) a learned, task-conditioned critic active during inference, and (iii) an endogenous gain controller that adaptively regulates sampling entropy during generation.

### 6.1 Pure prediction (decoder-only; no internal evaluation)

Autoregressive models trained for next-token prediction excel at fluent continuation but have no internal judge or regulator; behavior is largely governed by external decoding hyperparameters (e.g., temperature, top-*p*) (Sutskever et al., 2014). Regulated decoding can mitigate repetition or dullness but remains an external knob rather than an internalized policy (Holtzman et al., 2020). In GS-3 terms, this family has a generator but lacks an internal critic and lacks an endogenous gain controller.

### 6.2 Prompt-scaffolded reflectivity

Prompting can scaffold brief internal critique—e.g., chain-of-thought styles that elicit intermediate reasoning steps (Chu et al., 2024). These strategies often improve reliability on structured tasks yet remain scaffold-dependent: the “critic” is effectively encoded in the prompt template, not learned as a task-conditioned utility model. Exploration–exploitation is therefore not endogenously regulated, and adaptation across steps depends on the fixed script. In GS-3 terms, this family has a generator; the critic is externalized to prompts rather than learned and active during inference; there is no endogenous gain controller.

### 6.3 Retrieval-augmented generation

Coupling generation to a retriever injects external knowledge and improves factual grounding on knowledge-intensive tasks (Izacard and Grave, 2021). Standard retrieval-augmented generation (RAG) pipelines still lack a learned internal critic that scores candidate continuations for task utility and a gain policy that adapts search breadth in real time. Breadth is set by retrieval depth and decoding parameters rather than updated by a live utility signal. In GS-3 terms, this family has a generator but lacks an internal critic and an endogenous gain controller.

### 6.4 Plug-and-play steering at decoding time

Decoding-time “plug-and-play” controls can up- or down-weight attributes (e.g., sentiment, toxicity) on the fly without retraining the backbone (Pascual et al., 2021). Steering nudges the generator but does not maintain a persistent, task-conditioned evaluator nor an endogenous entropy controller tied to performance feedback. In GS-3 terms, this family has a generator but lacks an internal critic and an endogenous gain controller.

### 6.5 Instruction-following with human feedback

Instruction-tuned models align behavior with human preferences via feedback pipelines. Surveys and analyses detail data collection, objectives, and limitations of feedback-integrated NLG (Fernandes et al., 2023; Casper et al., 2024). These pipelines primarily externalize evaluation into the training data or reward modeling; at inference, most systems continue to rely on fixed decoding settings rather than a live gain policy tied to moment-to-moment utility. In GS-3 terms, this family has a generator; the critic is effectively baked in via training rather than active during inference; there is no endogenous gain controller.

### 6.6 Self-correction and iterative refinement

Test-time self-correction mechanisms iteratively propose, critique, and revise drafts. A recent survey maps when such loops help or fail across tasks (Kamoi et al., 2024), and domain-specific controllers demonstrate gains in code generation with explicit revise-and-retry cycles (Ding et al., 2024). However, loop structure and revision depth are typically hand-designed; the exploration–verification balance is not governed by an internal, learned gain signal that adapts step-to-step. In GS-3 terms, this family has a generator; the critic is scripted/self-referential rather than learned and general; there is no endogenous gain controller.

### 6.7 Multi-agent orchestration

Agentic set-ups coordinate multiple LLMs (planner/critic/worker roles), sometimes with memory and tools, to simulate social feedback dynamics (Park et al., 2023). While this can approximate a multi-perspective critique, policies are usually scripted; there is no single controller that adapts sampling entropy from reward-prediction error within a run. In GS-3 terms, this family has a generator; the critic role is scripted; there is no endogenous gain controller.

### 6.8 Human–AI co-creation and workflow integration

In professional settings, LLM support tends to increase throughput and fluency; without structured protocols, it can also reduce variety or drift from constraints (Chen and Chan, 2024; Chakrabarty et al., 2024). Effective workflows, therefore, need explicit mechanisms to preserve diversity while maintaining task fit—precisely the trade-off that GS-3 formalizes via a learned critic and adaptive gain.

### 6.9 Population-level effects and homogenization risk

At scale, generative assistance can raise individual originality while lowering collective diversity, indicating homogenization pressure when many users draw from similarly tuned models and prompts (Doshi and Hauser, 2024). GS-3’s evaluation emphasizes not only artifact-level usefulness and novelty but also across-run dispersion, making homogenization an explicit quantity to measure and manage via the gain policy and plural critics.

### 6.10 Conceptual status of LLM creativity

Debates continue on whether contemporary LLMs meet criteria for creativity, where limits remain, and how to evaluate claims (Franceschelli and Musolesi, 2024). GS-3 is positioned as a control-theoretic addition: it does not claim that steering, prompting, or retrieval alone are insufficient, but that creative competence requires an internalized evaluator and an adaptive regulator with falsifiable process-level signatures (e.g., alternating associative-distance density and adaptive verification rates).

### 6.11 What is still missing (gap analysis)

Across these families, three ingredients remain only partially addressed:

1. A learned, task-conditioned critic active during generation (not only at training time or via prompts).
2. An adaptive gain controller that smoothly adjusts sampling entropy from a simple learning signal within the session.
3. Process-level signatures (cycling in associative-distance density; adaptive verification rates) that make the mechanism auditable.
4. GS-3 contributes exactly these pieces while remaining architecture-agnostic and compatible with standard comparators (Holtzman et al., 2020; Izacard and Grave, 2021; Pascual et al., 2021; Chu et al., 2024).

### 6.12 Summary

Current systems achieve parts of the creative loop—fluent expansion, external steering, retrieval grounding, scripted reflection—but lack an endogenous, learned mechanism that coordinates expansion with evaluation under adaptive gain. GS-3 specifies that mechanism and its signatures, providing clear ablations and pass–fail criteria for empirical tests in Section 5.

## 7 Ethics, governance, and responsible deployment

GS-3 aims to operationalize creative generation while minimizing societal risk. This section outlines risks, design safeguards, reporting standards, and governance practices that make GS-3 auditable and alignable in real use.

### 7.1 Risk landscape

#### 7.1.1 Bias and preference overfitting

Training or validating critics on narrow rater groups can encode majority preferences and crowd out minority aesthetics. Surveys and analyses of feedback-driven NLG document how data collection, rater instructions, objective choice, and optimization targets shape model behavior and can entrench unwanted preferences (Fernandes et al., 2023; Casper et al., 2024).

#### 7.1.2 Homogenization

At the population level, assistance can raise individual originality while reducing collective diversity—consistent with convergent styles and “mode collapse” at scale (Doshi and Hauser, 2024). This risk is directly relevant to GS-3’s diversity objective.

#### 7.1.3 Scaffold dependence

Prompted self-reflection (e.g., chain-of-thought styles) can improve reliability on some tasks yet remains externally scaffolded and can fail outside its design envelope (Chu et al., 2024). GS-3 treats such reflectivity as a baseline, not a substitute for an internal critic and gain policy.

#### 7.1.4 Attribution and provenance

Use of external knowledge without source tracking can blur accountability. Retrieval-augmented generation highlights the need for explicit provenance trails (Izacard and Grave, 2021).

#### 7.1.5 Manipulation and reward gaming

Systems optimizing proxy rewards may learn to exploit engagement-like signals rather than usefulness; this motivates transparent utility functions, plural critics, and caps on entropy changes per cycle (Casper et al., 2024).

### 7.2 Design safeguards

#### 7.2.1 Plural critics and counterfactual scoring

Train multiple critics with diverse rater pools and aggregate via robust methods; monitor divergence to detect preference drift (Fernandes et al., 2023; Casper et al., 2024).

#### 7.2.2 Telemetry for audit

Log candidate sets, critic scores, temperature trajectory, retrieval queries and sources, and rationale snippets. Release redacted logs for external auditing subject to privacy constraints.

#### 7.2.3 Entropy governance

Enforce bounded-logistic gain (Section 4) with rate-limiters on temperature change per cycle; preregister *T*₍min₎, *T*₍max₎, and learning-rate bounds.

#### 7.2.4 Attribute controls with disclosure

When using decoding-time steering, employ plug-and-play controls that nudge attributes without retraining, and disclose active controls in outputs (Pascual et al., 2021).

#### 7.2.5 Knowledge provenance

For any grounded claim, attach sources returned by the retriever and prefer evidence-linked output modes (Izacard and Grave, 2021).

#### 7.2.6 Co-creation protocols

In collaborative settings, use structured prompts and rubrics to preserve variety and constraint adherence (Chen and Chan, 2024; Chakrabarty et al., 2024).

### 7.3 Reporting standards

#### 7.3.1 Preregistration

Publish prompts, success criteria, decoding budgets, and ablation plans.

#### 7.3.2 Human evaluation

Provide rater training materials and report inter-rater reliability; define task-conditioned rubrics. If using learned reward models, document data provenance, validation against held-out human judgments, and failure analyses (Fernandes et al., 2023; Casper et al., 2024).

#### 7.3.3 Process-level signatures

Report associative-distance density, analytic-verification ratio, and convergence latency with confidence intervals, plus spectral/auto-correlation analyses evidencing cycling.

#### 7.3.4 Release materials

Share code for metrics, ablation toggles, seeds and decoding settings, and (where possible) a minimal GS-3 implementation to reproduce tables and figures.

### 7.4 Governance and oversight

#### 7.4.1 Principle-guided constraints

Where high-stakes governance is required, adopt constitution-style rule sets derived from public input, and bind the critic’s utility and admissible entropy range to these principles (Huang et al., 2024). This layer is complementary to, not a replacement for, GS-3’s endogenous regulation.

#### 7.4.2 Independent review

Establish review boards to audit data governance, preference diversity, impact on stakeholders, and telemetry practices; publish periodic system cards summarizing risks and mitigations.

#### 7.4.3 User agency and consent

Provide clear affordances to decline data use for feedback, select preference profiles, and request provenance for retrieved evidence.

### 7.5 Boundary conditions

GS-3 is a control-theoretic proposal for creative generation, not a normative theory of cultural value. It does not by itself resolve questions of authorship or intellectual property; rather, it supplies the mechanisms and measurements by which such policies can be evaluated.

## 8 Discussion and open problems

This section synthesizes the argument, states boundary conditions, and outlines priority experiments that could support or falsify Generative System 3 (GS-3). Emphasis is on what the framework adds beyond existing accounts, where it may fail, and how to test it with published, auditable methods.

### 8.1 What GS-3 adds

GS-3 contributes a concrete control story for moving beyond fluent prediction and scaffolded reflectivity: a generator for associative expansion, a learned critic for task-conditioned appraisal, and an endogenous gain controller that adjusts sampling entropy online from a reward-prediction error. In contrast to externally tuned decoding (e.g., temperature sweeps, beam width), the exploration–exploitation balance becomes a learned, auditable policy with measurable signatures (Sections 4–5). This reorients evaluation from static artifacts to process-level observables and ablation tests using preregistered metrics and baselines (Holtzman et al., 2020; Chu et al., 2024; Izacard and Grave, 2021).

### 8.2 Boundary conditions and limitations

#### 8.2.1 Non-isomorphism

The DMN–CEN–dopamine mapping is a functional analogy, not a claim of biological identity. Neuromodulators shape integration/segregation and effort allocation in flexible cognition, but their roles are contextual and multifaceted (Shine, 2019; Westbrook et al., 2021). Temperature and related decoding controls are only rough proxies for gain in artificial systems.

#### 8.2.2 Task domain and priors

Gains from an endogenous controller will depend on task structure. Problems with tight constraints may benefit more from strong critics and narrower entropy; open-ended ideation may require wider entropy and more permissive critics. Long-horizon composition introduces additional stability–adaptation trade-offs (Section 4).

### 8.3 Proxy risks and evaluation pitfalls

#### 8.3.1 Preference models

Utility models trained from narrow rater pools can encode unwanted biases or collapse diversity; the literature on feedback-integrated NLG documents these risks and recommended safeguards (Fernandes et al., 2023; Casper et al., 2024). Accordingly, GS-3 advocates plural critics, provenance for feedback data, and validation of reward models against held-out human judgments.

#### 8.3.2 Measurement sensitivity

Novelty measured as embedding distance depends on the encoder and baseline corpus; conclusions should be cross-checked with human judgments and alternative encoders. Usefulness scores must report rater training and reliability; when model-based, they require external validation (Fernandes et al., 2023; Casper et al., 2024).

### 8.4 Decoding pathologies and controller claims

High temperature can increase diversity at the expense of coherence; low temperature can induce repetition and dullness—well-characterized failure modes under standard decoding (Holtzman et al., 2020). GS-3 does not claim these trade-offs disappear, but rather that an online gain policy can steer them adaptively within a run; this remains an empirical question addressed by the pass–fail criteria in Section 5.

### 8.5 Priority experiments

Minimal single-backbone implementations should be compared against regulated and reflective baselines under matched compute, prompts, and retrieval settings (Holtzman et al., 2020; Chu et al., 2024; Izacard and Grave, 2021). Divergent-thinking tasks (e.g., alternative uses), constrained creation (e.g., headlines with requirements), and long-horizon composition provide complementary stress tests. Writer-in-the-loop tasks probe fluency–variety trade-offs in professional workflows (Chen and Chan, 2024; Chakrabarty et al., 2024). At population scale, audits should test for homogenization (increases in individual usefulness/originality alongside decreases in collective diversity) and whether plural critics and gain policies mitigate it (Doshi and Hauser, 2024). Preregistration should include hypotheses, ablations (remove critic; freeze gain; swap policies), stopping rules, and telemetry (candidate sets, critic scores, reward-prediction error, temperature trajectory) to support external audit.

### 8.6 Open problems

#### 8.6.1 Multimodal and embodied extensions

Extending the generator–critic–gain loop to vision, audio, and action raises questions about shared versus modality-specific critics and controllers, especially for agents that learn from interaction.

#### 8.6.2 Memory and priors

How should the running baseline of expected utility be maintained across chapters, sessions, or projects without inducing inertia or overfitting to early successes?

#### 8.6.3 Plural critics and value alignment

Aggregating diverse preference models may preserve diversity while maintaining task fit, but it complicates optimization and governance (Fernandes et al., 2023; Casper et al., 2024). What aggregation rules best handle disagreement without masking minority values? Can constitution-style, publicly derived principles provide guardrails without collapsing variety (Huang et al., 2024)?

#### 8.6.4 Interaction with external tools

Retrieval and plug-and-play steering provide complementary control surfaces; their interaction with an internal gain policy requires systematic mapping to avoid redundant or destabilizing effects (Izacard and Grave, 2021; Pascual et al., 2021).

### 8.7 Summary

GS-3 is a proposal to turn a functional analogy into a testable engineering target. Its value hinges on rigorous comparisons to strong baselines, preregistered metrics and ablations, and transparent reporting. If its predictions fail, that outcome is informative—favoring alternative accounts such as scaffolded reflectivity or purely external regulation. If they succeed, they mark a step toward artificial systems that manage the tension between novelty, usefulness, and diversity by learning to regulate their own creative process (Holtzman et al., 2020; Chu et al., 2024; Izacard and Grave, 2021; Chen and Chan, 2024; Doshi and Hauser, 2024).
