# Supplementary material for: Artificial Creativity: from predictive AI to Generative System 3
Source: Front Artif Intell. 2025 Oct 15;8:1654716. doi: 10.3389/frai.2025.1654716 (PMC12568592; doi:10.3389/frai.2025.1654716)
Supplement: Supplementary file 1 [file Data_Sheet_1.PDF]

Supplementary Data Sheet 1. Formal details and pseudocode for GS-3

This file complements Section 4 of the main manuscript. It provides full formulas and illustrative pseudocode for baselines/signals, explicit novelty and diversity metrics, alternative gain policies with stability notes, and behavioral-index computations. Use "Algorithm S1 / Table S# / Figure S#" numbering when citing from the main text.

A.1 Baselines and signals

Exponentially weighted moving average (EWMA).

$\bar{U}_{(t+1)} = (1 - \beta)\cdot\bar{U}_{(t)} + \beta\cdot U_{(best,\ t)}$, with $\beta \in (0, 1]$. Initialize $\bar{U}_{(0)}$ by the first observed best score or a small constant.

Reward-prediction error.

$\delta_{(t)} = U_{(best,\ t)} - \bar{U}_{(t)}$.

A.2 Novelty and diversity (explicit formulas)

Nearest-neighbor novelty (cosine).

$N(x) = 1 - \max_{(b \in B)} \cos(e(x), e(b))$.

Here $e(\cdot)$ is a fixed embedding function and B is a preregistered baseline corpus for the task.

Across-run diversity (mean pairwise dispersion).

$D = 2/[r(r - 1)] \cdot \Sigma_{i<j} [1 - \cos(e(x_i), e(x_j))]$ for r independent runs of the same prompt.

A.3 Alternative gain policies and stability notes

Linear update.

$T_{(g,\ t+1)} = T_{(g,\ t)} + \kappa\cdot\delta_{(t)}$, with hard bounds $T_{(min)} \leq T_{(g)} \leq T_{(max)}$. Choose κ via a small sweep to avoid rapid saturation.

Exponential update.

$T_{(g,\ t+1)} = T_{(g,\ t)} \times \exp(\eta\cdot\delta_{(t)})$. Clip δ to $[-1, 1]$ and keep η small to limit per-step changes (e.g., $< 1.6\times$).

Sensitivity analysis.

Sweep η, β, and $[T_{(min)}, T_{(max)}]$ to chart novelty–usefulness–diversity trade-offs; report regions showing oscillatory $T_{(g)}$ or saturation.

A.4 Behavioral indices: computation details

Associative-distance density (ADD).

Compute cosine distance between successive idea units (e.g., sentences) within a run; summarize by distributional shape and periodicity (e.g., spectral power at low frequencies indicating alternation).

Analytic-verification ratio (AVR).

Count cycles in which C requests resampling at a lower $T_{(g)}$; divide by total cycles.

Convergence latency (CL).

Count cycles until a preregistered success threshold is met (e.g., rubric score $\geq \tau$).

A.5 Algorithm S1: GS-3 pseudocode (illustrative)

```
while task_not_finished:
    candidates = G(context, T_g, k)
    scores = C(candidates, context) # U(x | task, context)
    best = argmax(scores)
    delta = scores[best] - U_bar # U_bar corresponds to Ū
    context.append(candidates[best])
    T_g = T_min + (T_max - T_min) * sigmoid(alpha + eta * delta)
    U_bar = (1 - beta) * U_bar + beta * scores[best]
```

Notes. Choose a fixed encoder $e(\cdot)$ and baseline B in advance; preregister all prompts, bounds, and stopping rules. Report confidence intervals and effect sizes. When comparing against regulated and reflective baselines, equalize compute budgets and decoding constraints (Holtzman et al., 2020). Preference-trained policies and their rater procedures should be documented when used (Fernandes, Ribeiro, & Martins, 2023; Casper, Hadfield, & Leike, 2024). Logit steering or other decoding-time attribute controls, if applied, should be disclosed (Pascual, Egressy, Meister, Cotterell, & Wattenhofer, 2021).
